# Sarcopenia predicts cardiovascular disease in chronic kidney disease at advanced stage and associated risks

**DOI:** 10.1097/MD.0000000000035976

**Published:** 2023-11-10

**Authors:** Yingli Xuan, Shiqing Pang, Weizhen Xie, Ruibin He, Li Qin, Jiangzi Yuan

**Affiliations:** a Department of Nephrology, Baoshan Branch of Renji Hospital, School of Medicine Shanghai Jiao Tong University, Shanghai, China.

**Keywords:** cardiovascular diseases (CVD), chronic kidney disease (CKD), inflammation, sarcopenia

## Abstract

Chronic kidney disease (CKD) has been associated with a higher risk of cardiovascular disease (CVD), and sarcopenia is a new risk factor for CKD. However, whether sarcopenia predicts CVD in CKD remains to be determined. Sarcopenia would predict CVD in CKD at advanced stage. This analysis included 101 patients with CKD at stage 3 or over to determine the prevalence of sarcopenia and cardiovascular disease in patients with CKD at stage 3 or over in our center. The patients were further categorized into sarcopenia group (N = 19) and non-sarcopenia group (N = 82) according to the diagnostic criteria for sarcopenia. Data on demographics, laboratory tests, and measurements of extracardiac adipose tissue thickness (EAT) was collected. The prevalence of sarcopenia in patients with CKD at stage ≥ 3 was 19%. Compared with non-sarcopenia group, patients from the sarcopenia group were older (*P* = .005), and presented longer disease durations (*P* = .002). The serum level of albumin was significantly decreased, (*P* = .047), and high-sensitivity C-reactive protein level (CRP) was significantly increased (*P* = .003) in sarcopenia group. In addition, the EAT was thicker in the sarcopenia group compared with non-sarcopenia group (*P* = .032). Furthermore, the le-stratified atherosclerotic cardiovascular disease (ASCVD) risk scores were positively correlated with inflammation, nutrition, body mass index (BMI) and disease duration of CKD in sarcopenia group (*P* < .001). Patients with CKD are prone to have sacropenia, which is associated with inflammation and malnutrition. Presence of sarcopenia in CKD patients predicts the risk of ASCVD.

## 1. Introduction

Chronic kidney disease (CKD) has a high prevalence (10%–13% of the population), and is associated with a higher risk of cardiovascular disease (CVD) (more than half).^[1]^ However, the underlying mechanisms for higher prevalence of CVD in these patients still remains largely unknown.

Sarcopenia is a disorder featured by progressive and widespread loss of skeletal muscle mass and functions, and is associated with increased risk of adverse events such as physical decline, decreased quality of life or death.^[2]^ Age, gender, lifestyle as well as chronic disease such as diabetes and CKD have been considered as risk factors for sarcopenia.^[3–7]^ The prevalence of sarcopenia ranges between 5.9% and 9.8% in non-dialytic CKD patients,^[8]^ and increases to a higher prevalence of 29.3% or over in patients that underwent hemodialysis.^[9]^ Sarcopenia has been associated with poor physical performance^[10]^ and mortality^[11,12]^ in CKD. Previous study has shown that the disorder is associated with an increased risk of cardiovascular events in patients with CKD, and may subsequently reduce the ability for exercise during the fatigue period.^[13]^

Previous studies have shown a strong association between CKD and sarcopenia in the general population, suggesting that sarcopenia is a new risk factor for CKD.^[14]^ In addition, several studies have proposed sarcopenia as a new prognostic factor for cardiovascular disease. However, sarcopenia is not routinely assessed in clinical practice, and the relationship between the disease and markers for CVD in CKD patients, as well as the underlying mechanisms remain to be determined.^[15]^

The present study evaluated sarcopenia-related parameters and the risk of CVD in a cohort of CKD patients with disease stage ≥ 3.

## 2. Methods

### 2.1. Study design and participants

The study recruited 101 patients who were admitted between August 2019 and August 2022. Patients with CKD at stage 3 to 5d, including those came for hemodialysis (HD) or peritoneal dialysis, aged 18 to 80 years and signed informed consent were included in the study. The cohort consisted of 17 CKD patients at stage 3, 17 CKD patients at stage 4, and 67 CKD patients at stage 5. Patients with impairments in cognition, speech or hearing, acute cerebrovascular disease and advanced malignancy were excluded from the study. The enrolled patients who received dialysis were all managed by the procedures sufficiently. The study was conducted per the principles of the Declaration of Helsinki, and was approved by Ethics Committee Baoshan Branch of Ren Ji Hospital, School of Medicine, Shanghai Jiao Tong University(2019-qkwkt-004). Written informed consent was obtained from all patients.

### 2.2. Clinical parameters and biochemical analysis

The following demographics were collected from all participants: age, sex, height, weight, blood pressure, primary diseases, comorbidities, and lifestyle such as smoking and exercise (more than 3 times per week and more than half an hour each time). Fasting venous blood samples were collected to determine the levels of serum albumin, prealbumin, high-sensitivity C-reactive protein (hs-CRP), total cholesterol, triglycerides, low-density lipoprotein cholesterol, high-density lipoprotein cholesterol, fasting blood glucose, glycated hemoglobin, blood creatinine, plasma brain natriuretic peptide (BNP), parathyroid hormone, calcium, phosphorus, and 25-hydroxy (OH) vitamin D. Body mass index (BMI) was calculated as follows: BMI = weight/square of height (kg/m^2^). Patients who received HD were assessed during the middle of the week, while those received peritoneal dialysis were evaluated in the morning before the first replacement, when presenting an empty peritoneum.

### 2.3. Screening for sarcopenia

Sarcopenia was diagnosed according to the recommendations of the Asian Working Group for Sarcopenia, which defined the disease as age-related loss of muscle mass, plus low muscle strength, and/or low physical performance.^[16]^ Sarcopenia was diagnosed when 2 or more of the following criteria were met: Lean tissue index (LTI) < 7.0 kg/m^2^ for male or LTI < 5.7 kg/m^2^ for female. A body composition monitor (Fresenius Medical Care, Germany) was used to determine the LTI of the individual. The participant lay flat for 5 minutes, with limbs separated naturally. The electrode was placed on ipsilateral metacarpal, wrist, metatarsal toe, and ankle joint. The distance between adjacent electrodes was at least 3 cm, and a wire was used for measurement. Handgrip strength (HGS) < 26 kg for male and < 18 kg for female. The HGS was determined using an EH101 electronic gripmeter with a domestic camera. HGS of the dominant hand was measured 3 times, and the maximum measurement was recorded. Walking speed ≤ 0.8 m/s. The average walking speed was calculated by asking the participant to walk within a test area of 4m at a conventional walking speed.^[17]^

### 2.4. Assessment of extracardial adipose tissue thickness and CVD events

We evaluated extracardial adipose tissue (EAT) as a marker to assess risk of CVD. Color Doppler ultrasound was performed by a full-time cardiac ultrasound doctor at the hospital. S4 probe and M-type methods were applied to measure EAT thickness. The EAT thickness was determined by measuring the vertical thickness of the free wall of the right ventricle at the end of cardiac diastole for 3 cardiac cycles under in the long sternum axis view of 3 cardiac cycles. CVD events were documented from patients’ medical history, including myocardial infarction, coronary artery bypass surgery or percutaneous coronary intervention, congestive heart failure, angina pectoris, cerebrovascular diseases, and peripheral vascular diseases.

### 2.5. Risk assessment for atherosclerotic cardiovascular disease (ASCVD)

Risk for ASCVD was evaluated using the ASCVD risk score from the 15th National Public Relations Task Force, which is a simple tool assessing the risk for ischemic cardiovascular disease in Chinese. Six risk factors, including age, blood pressure, BMI, total serum cholesterol, diabetes, and smoking, were scored independently, and subsequently summed up to estimate the risk for ASCVD in the next 10 years. Exercise and lifestyles were also considered.

### 2.6. Statistical analysis

Statistical analysis was performed using SPSS (IBM, Armonk, NY, version 22.0 for Windows). Continuous and categorical variables were expressed as mean ± SD and numbers (including percentage), respectively. Statistical significance was determined if *P* value is <.05. The demographics and related measurements were compared between groups using one-way analysis of variance for continuous variables and ×2 tests for categorical variables, followed by post hoc analyses using the Bonferroni method. The association between sarcopenia and ASCVD risk score was evaluated using χ^2^ test after transforming variables into quartiles. Multivariate logistic regression analysis was applied to determine the independent association between sarcopenia and high ASCVD risk after adjusting for age and sex in Model 1. Exercise and smoking status were adjusted additionally in model 2. Hypertension, type 2 diabetes (T2D), obesity, and hypercholesterolemia were further additionally adjusted in Model 3. To assess the impact of high ASCVD risk, adjusted Model 3 was applied to each factor, including sarcopenia, hypertension, T2D, smoking, exercise, and hypercholesterolemia.

## 3. Results

### 3.1. Demographics, laboratory and imaging assessments

A total of 101patients were recruited. The prevalence of sarcopenia was 19% in this cohort. Patients were subsequently divided into sarcopenia group (N = 19) and non-sarcopenia group (N = 82) according to the criteria for sarcopenia. Patients in the sarcopenia group as well as sarcopenia patients at CKD stage 5 were significantly older (*P* = .006; *P* = .001), and were more easily to suffer from comorbidities of CVD (*P* = .021; *P* = .034). In addition, sarcopenia patients also presented a longer duration of CKD (*P* = .002). The circulating level of hs-CRP was significantly higher (*P* = .003) in the sacropenia group than that in the non-sarcopenia group. In contrast, the serum level of albumin was lower (*P* = .047) in the sacropenia group compared with non-sarcopenia group. Ultrasonic examination showed that EAT significantly expanded in the sarcopenia group and sarcopenia patients at CKD stage 5 compared to those in the non-sarcopenia group (*P* = .032; *P* = .001) (Table 1). The dialysis vintage for patients that underwent hemodialysis was 2.05 ± 0.72 years in sarcopenia group, while was 1.64 ± 0.56 years in non-sarcopenia group.

**Table 1:** Demographics, laboratory and imaging characteristics.

	Sarcopenia		Non-Sarcopenia		*P* value	
	Total (N = 19)	CKD stage 5	Total (N = 82)	CKD stage 5	Total	CKD stage 5
Age	72.29 ± 9.18	69.57 ± 10.24	53.58 ± 13.81	51.26 ± 13.08	0.006*	0.001*
Female/Male n(%)	10(55)/8 (44)	4 (28)/10 (72)	42 (51)/40 (48)	18 (34)/35 (66)	2.345	0.702
BMI(kg/m^2^)	24.43 ± 3.42	22.47 ± 3.03	22.01 ± 2.42	22.91 ± 3.23	0.057	0.648
SBP(mm Hg)	141 ± 14.10	132.78 ± 15.56	143 ± 11.46	140.62 ± 16.16	3.789	0.109
DBP(mm Hg)	74 ± 5.3	76.21 ± 11.62	80 ± 7.4	83.30 ± 12.06	3.597	0.053
Duration of chronic kidney disease (years)	18 ± 4		9 ± 3		0.002*	
Current smoker n(%)	11 (58)	6 (42)	46 (56)	21 (39)	3.412	0.826
Exercise n(%)	10 (52)	5 (35)	38 (46)	17 (32)	3.567	0.797
Primary disease						
Diabetes n(%)	6(33)	4(28)	30(36)	20(37)	2.675	0.695
Renal damage due to primary hypertension n(%)	4(22)	3(21)	15(18)	10(18)	3.497	0.487
Primary glomerulonephritisn n(%)	8(44)	7(50)	34(41)	20(37)	3.567	0.477
Others	0 (0)	0 (0)	3 (3)	3 (5)	1.076	1.006
Comorbidities						
Diabetes n(%)	12(63)	10(71)	38(46)	25(47)	1.123	1.223
Obesity n(%)	9 (20)	6 (42)	14 (17)	11 (20)	0.069	0.079
CVD cardiovascular disease n(%)	18(100)	14(100)	40(48)	39(73)	0.021*	0.034*
ASCVD risk (%) in 10 years	55		30		<0.001*	
Albumin (g/L)	29.89 ± 3.03	35.65 ± 7.21	35.93 ± 4.91	34.62 ± 6.95	0.047*	0.629
Prealbumin (mg/L)	302.76 ± 69.50	243.88 ± 104.67	333.58 ± 58.28	292.11 ± 86.53	0.314	0.081
CRP(mg/L)	5.27 ± 4.80	6.87 ± 9.03	0.61 ± 0.32	6.65 ± 21.98	0.003*	0.971
Cholesterol (mmol/L)	3.85 ± 0.7	3.72 ± 1.02	4.21 ± 1.44	3.55 ± 1.35	0.053	0.074
Low density lipoprotein (mmol/L)	3.22 ± 0.92	2.04 ± 0.66	3.20 ± 1.31	2.50 ± 0.90	0.970	0.083
HbA1c(%)	6.27 ± 0.64	5.37 ± 0.64	5.98 ± 0.41	5.85 ± 0.65	0.244	0.959
Creatinine(µmol/L)	559 ± 22.4	527.24 ± 298.99	472 ± 23.9	534.60 ± 240.58	0.875	0.126
GFR(mL/min)	10 ± 2.1	9.65 ± 3.11	12 ± 2.2	9.12 ± 4.82	0.567	0.646
BNP(pg/mL)	179.42 ± 54.72	211.20 ± 146.67	229.75 ± 250.99	220.69 ± 130.12	0.516	0.976
PTH(ng/L)	136.07 ± 101.61	235.47 ± 212.19	127.64 ± 106.94	234.91 ± 144.24	0.868	0.418
Calcium(mmol/l)	2.23 ± 0.15	2.13 ± 0.22	2.27 ± 0.24	2.11 ± 0.32	0.725	0.817
Phosphorus(mmol/L)	1.47 ± 0.24	1.51 ± 0.38	1.51 ± 0.39	1.79 ± 0.48	0.828	0.046*
Vitamin D(mg/L)	7.57 ± 2.64	8.71 ± 5.68	8.25 ± 2.9	9.71 ± 4.39	0.618	0.454
EAT(mm)	5.91 ± 2.13	5.57 ± 1.10	2.51 ± 1.69	3.78 ± 1.21	0.032*	0.001*

BMI = body mass index, BNP = B-type natriuretic peptide, CRP = C-reactive protein, CVD = cardiovascular disease, DBP = diastolic blood pressure, EAT = extracardial adipose tissue, GFR = glomerular filtration rate, HbA1c = glycated hemoglobin, PTH = parathyroid hormone, SBP = systolic blood pressure.

* *P* < .05.

### 3.2. The relative risk of ASCVD according to sarcopenia status

**Figure 1.**
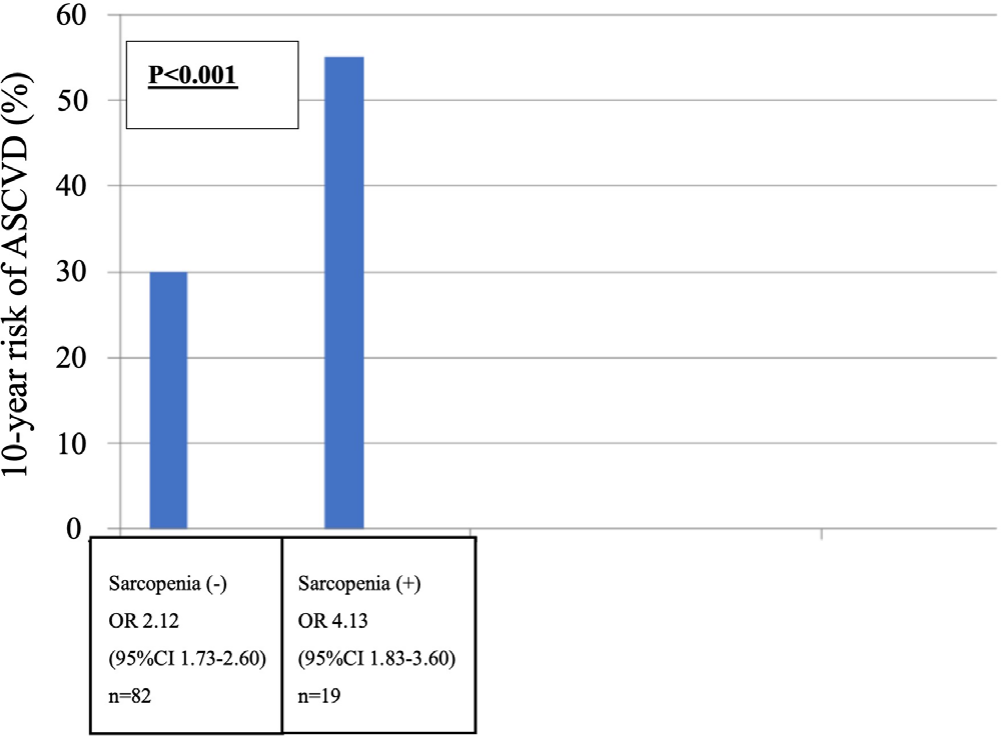
Risk of ASCVD in 10 yr. The probability of ASCVD in 10 yr was significantly higher in patients with sarcopenia compared with those without sarcopenia (*P* < .001). ASCVD = atherosclerotic cardiovascular disease, CI = confidence interval, OR = odds ratio.

Patients with sarcopenia presented significantly increased prevalence of high probability of ASCVD compared to non-sarcopenia patients (55% vs 30%; odds ratio (OR) = 4.13, 95% confidence interval (CI) = 1.83–3.60 among patients with sarcopenia vs OR = 2.12, 95% CI = 1.73–2.60 among patients without sarcopenia) (*P* < .001) (Fig. 1).

### 3.3. Association between high probability of ASCVD and the presence of sarcopenia

The association between high probability of ASCVD and sarcopenia after multistep adjustments is shown in Table 2. The relative risk for a high probability of ASCVD was assessed after sufficient adjustment (Model 3). Patients in the sarcopenia group presented a significantly higher OR, indicating a higher probability of ASCVD (OR = 1.72, *P* = .015) than patients in the non-sarcopenia group (used as a control group).

**Table 2:** Probability in ASCVD risk (>10%) in sarcopenia group and non-sarcopenia group

Multivariate models	Non-sarcopenia N = 82	Sarcopenia (OR, 95%CI, *P* value) N = 19
Model 1	1.00 (ref.)	3.30 2.39–4.48 *P* < .001
Model 2	1.00 (ref.)	4.12 3.04–5.59 *P* < .001
Model 3	1.00 (ref.)	1.63 1.13–1.96 *P* = .026

Model 1: adjusted for age and sex.

Model 2: adjusted for age, sex, exercise, and current smoking.

Model 3: adjusted for age, sex, hypertension, diabetes, obesity, hypercholesterolemia and creatine.

ASCVD = atherosclerotic cardiovascular disease, CI = confidence interval, CKD = chronic kidney disease, OR = odds ratio.

### 3.4. Associations between sarcopenia and cardiometabolic risk factors

Among varying cardiometabolic risk factors for ASCVD, we investigated the associations between sarcopenia and cardiometabolic risk factors with minimal adjustment for age, sex, smoking, and exercise (Table 3). Patients without sarcopenia were included in the control group. The risks for obesity (OR = 4.36), hypertension (OR = 3.88), T2D (OR = 3.62), and hypercholesterolemia (OR = 3.15) were increased in sarcopenia group (all *P* < .001). Patients in the sarcopenia group presented a much higher OR for obesity (OR = 4.36) than those in the control group (*P* < .001).

**Table 3:** Cardiometabolic risk factors in sarcopenia group and non-sarcopenia group.

ASCVD risk factors	NON-sarcopenia N = 82	Sarcopenia N = 19
Obesity	1.00 (ref.)	4.36 3.48–5.50 *P* < .001
Hypertension	1.00 (ref.)	3.88 2.67–4.42 *P* < .001
Diabetes	1.00 (ref.)	3.62 4.47–7.28 *P* < .001
Hypercholesterolemia	1.00 (ref.)	3.15 2.45–3.54 *P* < .001

Obesity was defined as BMI > 25 kg/m^2^; Hypercholesterolemia was defined as serum cholesterol > 5.72 mmol/L or taking cholesterol-lowering agents.

Adjusted for age, sex, smoking, and exercise. ASCVD = atherosclerotic cardiovascular disease, BMI = body mass index, CI = confidence interval, CKD = chronic kidney disease.

## 4. Discussion

In the present study, the prevalence of sarcopenia in CKD patients at stage 3 or higher is 19%. Patients with sacropenia are older, and have a longer disease duration for CKD. CKD and sarcopenia are both age-related diseases, with the human body beginning to lose muscle at a rate of 1% per year from the age of 30. In addition, the muscle mass of the elderly aged over 60 years decreases by 5–13%. The prevalence of sacropenia is as high as 50% in patients over 80 years old.^[18]^ Decreased muscle mass and functions are more common in CKD patients. The prevalence of sarcopenia in CKD patients absent from dialysis ranges from 6% to 10%, and the prevalence of sarcopenia or muscle atrophy in patients with end-stage renal disease ranges from 20% to 44%.^[19]^ The prevalence of sarcopenia in patients undergoing dialysis is approximately 50%. Progression of muscle atrophy is accompanied with decline in kidney functions.^[20]^ Proposed mechanisms for sarcopenia include endocrine hormone disorders, metabolic acidosis, chronic inflammation, uremic toxin accumulation, elevated level of angiotensin II, hypertension, poor blood glucose control, insulin resistance, nutritional deficiency, mineral bone diseases, edema, and other pathological conditions, resulting in exceeding of protein decomposition over protein synthesis and impaired ability for muscle precursor cells to proliferate and differentiate into muscle cells.^[21]^ Furthermore, the growth of muscle fibers is prohibited, while fibrosis of muscle tissue is induced, eventually leading to muscle attenuation.^[22]^

We also revealed that the plasma level of hs-CRP was increased and albumin level was decreased in the sarcopenia group, consistent with previous studies.^[23]^ Oxidative stress and decreased glomerular filtration rate has been suggested to lead to activation of inflammation in patients with CKD, and the levels of CRP, Interleukin-4 (IL-4), IL6(IL-6) and tumor necrosis factor (TNF-α) were increased in the circulation, resulting in imbalance between protein synthesis and degradation by inhibition of insulin-like growth factor-1 (Igf-1) signal transduction and activation of the ubiquitin-proteasome system (UPS) or CASPASE-3 proteasome pathways, which contribute to depletion of muscle protein.^[24]^ In addition, inflammation may induce microvascular impairments related to protein metabolism in muscles.^[25]^ Proteins play an essential role in maintaining the mass and strength of muscels.^[26]^ Castaneda et al found that elderly women who took a low-protein diet every day had a negative nitrogen balance presented as decreased in lean body mass and muscle functions, which is in contrast to presentations of individuals on an adequate protein diet. Another study showed that patients who developed end-stage renal disease and started with HD presented deteriorated nutritional parameters, trends of decrease in weight, BMI and fat mass, as well as increase in pro-inflammatory cytokine markers such as CRP and IL-6.^[27]^ Furthermore, the level of albumin was negatively correlated with pro-inflammatory status.^[28]^ The protein content of the elderly was lower, and the nutritional status was poorer in the sarcopenia group, which also increased the readmission rate.

The mechanism underlying the association between sarcopenia and cardiovascular disease has not been fully elucidated. The present study revealedthat the EAT expanded in the sacropenia group. EAT is composed of adipocytes and inflammatory cells, as well as stromal vessels or immune cells.^[29]^ The adipose tissue, dominated by white adipose tissue, contains macrophages and T lymphocytes that can promote the synthesis of active adipocytokines. There is no fibrous fascia layer between the myocardium and surrounding myocardium. Adipocytes produce a large number of inflammatory mediators that can penetrate myocardial fibers, which directly leads to inflammation around the coronary arteries and proliferation of smooth muscle cells around the coronary artery wall. Epicardial adipose tissue has a value that other adipose tissues do not have in predicting cardiovascular diseases.

We also assessed the risk of ASCVD according to the 15th National Public Relations Task Force in this cohort,^[30]^ which is a simple tool for the risk assessment of ischemic cardiovascular disease in China. We found that the prevalence of ASCVD was significantly higher in patients with sarcopenia than in those without. Studies have shown that sarcopenia is also a risk factor for cardiovascular disease, and may act by enhancing atrial stiffness, increasing chronic inflammation or reducing physical activity.^[31]^ Patients with older age, obesity, diabetes, hypertension, and dyslipidemia are more susceptible to sarcopenia, and these are also risk factors for CVD. These comorbidities frequently coexist in patients with CKD and sarcopenia. In this cross-sectional study, we could not assess the dynamic association between changes in CKD status and effects of interventions of sarcopenia on improving ASCVD events longitudinally. In addition, we were also unable to determine whether the effects of therapeutic interventions, such as lifestyle modification, exercise, weight loss, medications, nutritional support and protein supplements could improve manifestations of sarcopenia and reduce the risk of ASCVD. Well-designed, prospective, and longitudinal studies are warranted to explore the complex relationship and develop appropriate therapeutic interventions for CKD, sarcopenia, as well as to reduce cardiovascular risk. Nevertheless, our results showed the need for screening for sacropenia in CKD patients in clinical practice to allow possible interventions for ASCVD.

In conclusion, sarcopenia, independent of other metabolic and clinical factors in CKD patients, is significantly associated with increased risk for ASCVD in patients with CKD. Physicians should evaluate the status of sarcopenia and provide appropriate therapeutic interventions to reduce the risk of cardiovascular events by managing sarcopenia in patients with CKD.

## Author contributions

**Conceptualization:** Yingli Xuan, Weizhen Xie, Ruibin He, Jiangzi Yuan, Shiqing Pang.

**Supervision:** Jiangzi Yuan.

**Writing – original draft:** Li Qin.

**Writing – review & editing:** Yingli Xuan, Shiqing Pang, Weizhen Xie, Ruibin He, Li Qin, Jiangzi Yuan.
